# YAP in pancreatic cancer: oncogenic role and therapeutic strategy

**DOI:** 10.7150/thno.53438

**Published:** 2021-01-01

**Authors:** Wenhao Mao, Jia Mai, Hui Peng, Junhu Wan, Ting Sun

**Affiliations:** 1 Department of Clinical Laboratory, The First Affiliated Hospital of Zhengzhou University, Zhengzhou 450052, China.; 2 Department of Laboratory Medicine, West China Second Hospital, Sichuan University, Chengdu 610041, China.

**Keywords:** Pancreatic cancer, Hippo pathway, YAP, KRAS, therapeutic target

## Abstract

Pancreatic cancer, especially pancreatic ductal adenocarcinoma (PDAC), remains a fatal disease with few efficacious treatments. The Hippo signaling pathway, an evolutionarily conserved signaling module, plays critical roles in tissue homeostasis, organ size control and tumorigenesis. The transcriptional coactivator yes-associated protein (YAP), a major downstream effector of the Hippo pathway, is associated with various human cancers including PDAC. Considering its importance in cancer, YAP is emerging as a promising therapeutic target. In this review, we summarize the current understanding of the oncogenic role and regulatory mechanism of YAP in PDAC, and the potential therapeutic strategies targeting YAP.

## Introduction

Pancreatic cancer, of which 90% is attributable to pancreatic ductal adenocarcinoma (PDAC), remains the most lethal human malignancy, with an overall 5-year survival rate of 9% and a median survival of ~6 months 1, 2. Because of the aggressive nature and difficulty in early diagnosis, greater than one-half of pancreatic cancer patients are typically diagnosed at an advanced stage, for which the 5-year survival rate is only 3% 1. Despite new insights into the molecular mechanism of pancreatic cancer and advances in surgery, adjuvant therapy and chemotherapy, the overall prognosis of pancreatic cancer has not significantly improved. New approaches for the diagnosis and therapy of pancreatic cancer are urgently needed. Elucidating the signaling networks that regulate the development and progression of pancreatic cancer is critical to prevent or treat this lethal disease.

Yes-associated protein (YAP) and its paralog transcriptional co‑activator with PDZ-binding motif (TAZ) are the major downstream effectors of Hippo pathway 3. Although TAZ and YAP share similar structures and display a degree of functional redundancy, YAP has been studied more extensively. YAP is increasingly identified as a potent oncogene 3 and its abundance and activity are frequently increased in many cancers 4-12. In this review, we will comprehensively summarize the current studies of YAP in pancreatic cancer. Further understanding of the functions and regulatory mechanism of YAP is of great value for the prevention, early diagnosis and treatment of PDAC.

## The core of the Hippo pathway

**Figure 1:**
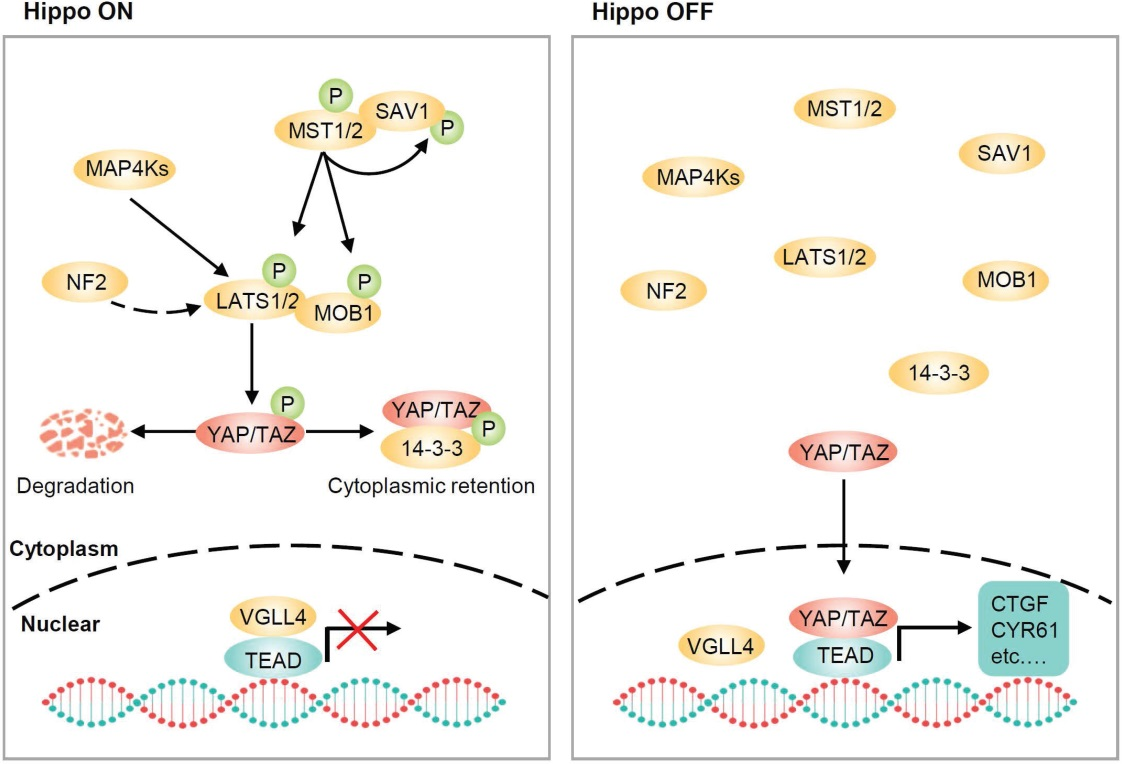
** Core components of the Hippo pathway in mammalian cells.** The core components of Hippo pathway consist of MST1/2, LATS1/2 and YAP/TAZ. When the Hippo pathway is ON, MST1/2, in complex with SAV1, phosphorylate LATS1/2 and MOB1. MAP4Ks also phosphorylate and activate LATS1/2. NF2 is an additional and potent activator of LATS1/2 that is devoid of kinase activity. Activated LATS1/2 phosphorylate YAP and TAZ, resulting in 14-3-3-mediated YAP/TAZ cytoplasmic retention and ubiquitin-mediated proteasomal degradation. When the Hippo pathway is OFF, YAP/TAZ are dephosphorylated and translocate to the nucleus, where they compete with VGLL4 for TEADs binding and induce the transcription of downstream target genes, such as CTGF and CYR61, which are involved in growth, proliferation, and survival.

The Hippo pathway, originally identified in* Drosophila*, is highly conserved in mammals and is important for organ size control, tissue homeostasis and regeneration 13-21. The core of the Hippo pathway in mammals consists of a kinase cascade, mammalian Ste20-like kinases 1/2 (MST1/2), large tumor suppressor 1/2 (LATS1/2), and transcriptional coactivators YAP and TAZ (**Figure 1**). MST1/2 form heterodimers with the adaptor protein SAV1, which functions as a partner of MST1/2 in promoting LATS1/2 phosphorylation 22. RASSF1A interacts with MST1/2 and induces their autophosphorylation and activation 23. MST1/2 then directly phosphorylates LATS1/2 and the cofactor MOB1. Phosphorylated LATS1/2 enhances autophosphorylation at their activation loop and phosphorylated MOB1 binds to the autoinhibitory domain of LATS1/2, leading to full activation 22, 24. NF2 is another potent activator of LATS1/2, and NF2 can directly interact with LATS1/2 and recruit them to the plasma membrane for activation by MST1/2 25, 26. In parallel to MST1/2, members of the MAP4K family, including MAP4K1/2/3/5 and MAP4K4/6/7 can also directly phosphorylate LATS1/2 at their hydrophobic motifs, resulting in LATS1/2 activation 27-29. Notably, TAO kinases have been found to act both upstream of and in parallel to MSTs to directly phosphorylate LATSs, raising the possibility that MST1/2 are not absolutely required for the regulation of LATS1/2 30, 31. Once activated, LATS1/2 directly phosphorylates YAP/TAZ and phosphorylated YAP/TAZ are sequestered in the cytoplasm by binding to 14-3-3 32-34. Moreover, subsequent phosphorylation of YAP/TAZ by CK1 leads to SCF β-TrCP-mediated ubiquitination and proteasomal degradation 35, 36. When LATS1/2 are inactive, dephosphorylated YAP/TAZ translocate to the nucleus, where they compete with VGLL4 (vestigial-like protein 4) and initiate gene transcription by interacting with transcription factors, particularly TEA domain (TEAD) family members 37-39. It is worth noting that the regulation of Hippo pathway is not static but rather dynamic, and this dynamic regulation leads to YAP rapidly shuttling between the cytoplasm and the nucleus 40.

## The posttranslational modifications of YAP

**Figure 2:**
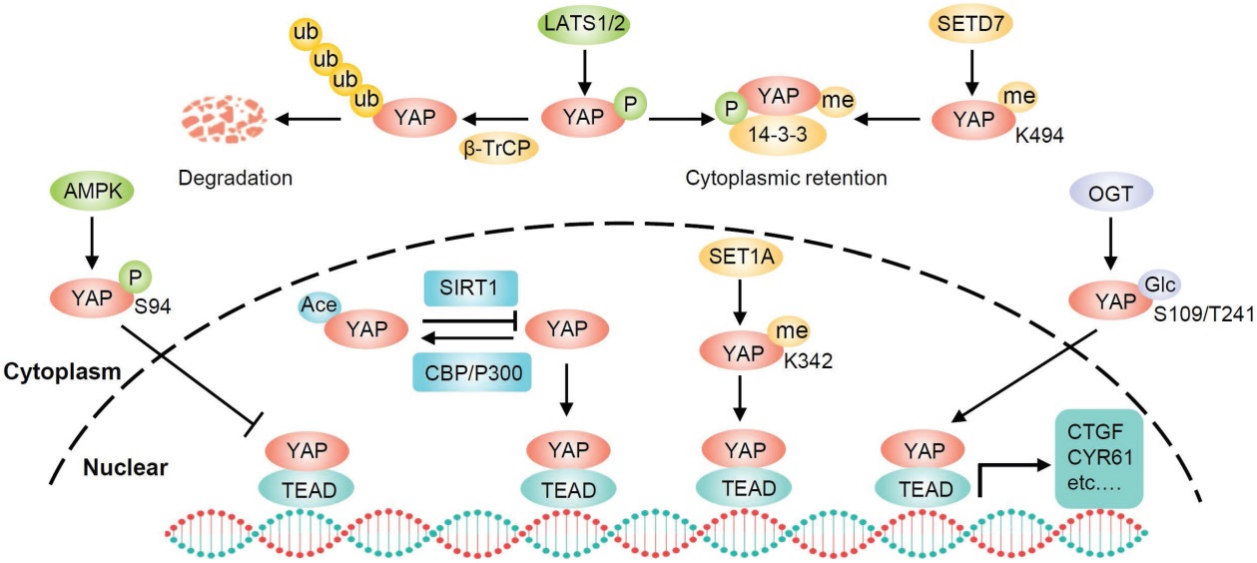
** Schematic illustration of the posttranslational modifications of YAP.** In addition to phosphorylation and ubiquitylation, YAP can also be regulated by several other posttranslational modifications including high glucose-stimulated YAP O-GlcNAcylation for increasing YAP stability, SIRT1-mediated YAP deacetylation for enhancing YAP/TEAD association, SETD7 methylated methylation for YAP cytoplasmic retention, and SET1A-mediated methylation for YAP nuclear retention.

Phosphorylation is the most common posttranslational modification of YAP and LATS1/2 are the primary upstream kinases. Moreover, AMPK can also directly phosphorylate YAP on several residues including the S61 and S94. Phosphorylation of YAP S94 by AMPK disrupts its interaction with TEAD and inhibits YAP function 41. However, exactly how AMPK-dependent S61 phosphorylation inhibits YAP transcriptional activity remains unknown 42. In addition to phosphorylation, YAP activity can be regulated through several other posttranslational modifications, including O-GlcNAcylation, acetylation, and methylation (**Figure 2**). When glucose is sufficient, YAP is O-GlcNAcylated at serine 109 or threonine 241 by O-GlcNAc transferase (OGT). YAP O-GlcNAcylation activates its transcriptional activity by disrupting the LATS-mediated phosphorylation and blocking its ubiquitination-mediated degradation by β-TrCP 43, 44. SIRT1-mediated deacetylation of YAP enhances the YAP/TEAD4 association and transcriptional activity, whereas acetyltransferases CBP and p300 are responsible for YAP acetylation 45, 46. Furthermore, SETD7 methylates YAP on lysine 494, and this methylation specifically promotes the cytoplasmic retention of YAP 47. Conversely, SET1A-mediated methylation of YAP at lysine 342 is essential for the nuclear retention of YAP 48. Therefore, YAP can be regulated by diverse posttranslational mechanisms.

## YAP and cancer metabolism

One hallmark of cancer cells is metabolic reprogramming. Significant progress has been made in the crosstalk between cancer metabolism and YAP signaling. Studies have shown that YAP is regulated by cellular energy stress. Both LATS kinase and AMPK were activated during glucose starvation, resulting in phosphorylation of YAP and contributing to its inactivation 42, 49. Besides, glycolysis is required to sustain YAP function, and the key enzyme of glycolysis, phosphofructokinase (PFK1), can bind to TEADs and promote their functional and biochemical cooperation with YAP 50. On the other hand, YAP reprograms glucose metabolism. YAP promoted glucose metabolism through transcriptional upregulation of glucose-transporter 3 (GLUT3) expression 42. In addition, YAP promoted glycolysis through upregulating the expression of lncRNA BCAR4, which subsequently coordinates the Hedgehog pathway to enhance the transcription of glycolysis activators HK2 and PFKFB3 51. Mitochondria are the powerhouses of the cell crucial for both anabolic and catabolic metabolism. The mitochondrial deoxyguanosine kinase (DGUOK) is a rate-limiting enzyme in the mitochondrial deoxynucleoside salvage pathway. DGUOK was found to inhibit mitochondrial respiration and self-renewal of lung cancer stem-like cells through preventing AMPK-mediated phosphorylation of YAP 52. Activation of lipid biosynthesis, is a major event in the metabolic transformation of cancer 53. Stearoyl-CoA-desaturase 1 (SCD1), the enzyme involved in fatty acids synthesis, is a key regulator of YAP in lung cancer stem cells through its involvement in wnt/β-catenin pathway 54.

In pancreatic cancer, activating mutation in *Kras* is the most prominent driver, present in over 90% of PDAC 55, 56. Kras-induced metabolic reprogramming is essential for pancreatic cancer cells to survive the environmental and nutrient stresses. As the downstream target of KRAS, YAP cooperates with Myc to maintain the global transcription of metabolic genes and metabolic homeostasis in Kras-driven PDAC 57. Notably, some drugs targeting metabolic regulation of YAP, such as metformin and statins, have been found to be potential treatment strategies for pancreatic cancer.

## The functional role of YAP in pancreatic cancer

Studies have indicated that YAP is overexpressed in PDAC and active YAP promotes pancreatic cancer cell proliferation, survival and metastasis 58, 59. Moreover, YAP has been identified as an independent prognostic marker of PDAC, and elevated YAP expression is associated with poor prognosis 60-62. In mouse models of pancreatic duct cell-specific deletion of Lats1/2, YAP cooperates with AP-1 to initiate pancreatic cancer development from ductal cells 63. Acinar cell-specific Lats1/2 deletions caused pancreatic inflammation and fibrosis are YAP dependent in adult mice 64. Moreover, YAP is essential for PDAC progression in *Kras*-mutant mice 65, 66. Zhang et al. found that, although YAP is dispensable for acinar to ductal metaplasia (ADM), an initial step in the progression to PDAC, YAP is essential for PanIN progression to PDAC 65. Gruber et al. found that YAP is required for the induction of ADM and progression to PanIN by direct up-regulation of JAK-STAT3 signaling 66. Mutant GNAS, as an onco-modulator of Kras-induced neoplasia, causes phosphorylated YAP to be sequestered in the cytoplasm and alters tumor progression 67. In addition, oncogenic activation of YAP collaborates with heterozygous *Kras*^MUT^ in driving PDAC tumorigenesis 68. Besides, YAP is required for cancer recurrence in the absence of KRAS 69. Recent studies have also demonstrated that YAP is a major driver of squamous subtype PDAC, independent of oncogenic KRAS 70. These findings raise an important insight that YAP not only acts as a PDAC driver downstream of KRAS but also enables PDAC to escape KRAS dependence 65, 69, 71.

**Figure 3:**
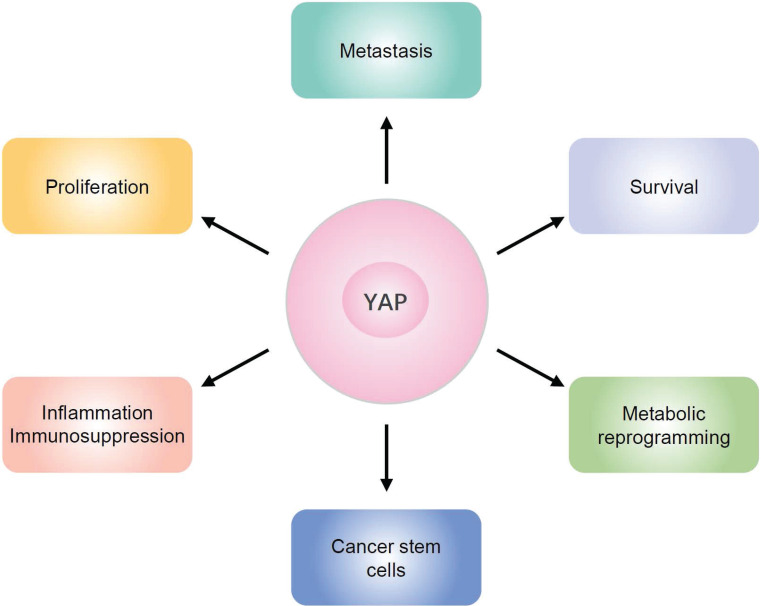
** Schematic overview of YAP functions in pancreatic cancer.** YAP exerts oncogenic functions in different aspects of PDAC, including proliferation, survival, metastasis, metabolic reprogramming, and cancer stem cells, as well as inflammation and immunosuppression.

YAP exerts oncogenic functions in different aspects of PDAC (**Figure 3**). YAP is essential to maintain the metabolic homeostasis in Kras-driven PDAC 57. Severe inflammation and profound immune suppression are common features of PDAC. Studies have demonstrated that YAP acts as a transcriptional driver of multiple cytokines, which in turn promote the differentiation and accumulation of myeloid-derived suppressor cells (MDSCs), and contributes to the strong immunosuppressive microenvironment in both mouse and human PDAC 72. Cancer stem cells (CSCs), which express stem cell marker CD133, have the ability to self-renew. Recent studies have demonstrated that YAP is downstream of HMGB1-TLR2 signaling to induce CD133^-^ cancer cell dedifferentiation and enhance pancreatic cancer stemness 73. YAP overexpression in PDAC induces NMU expression and promotes cell motility and tumor metastasis via upregulation of epithelial-mesenchymal transition (EMT) 62. Moreover, YAP also contributes to pancreatic cancer cell invasion and migration by disrupting tumor-stromal interactions 74.

## Regulation of YAP in PDAC

**Figure 4:**
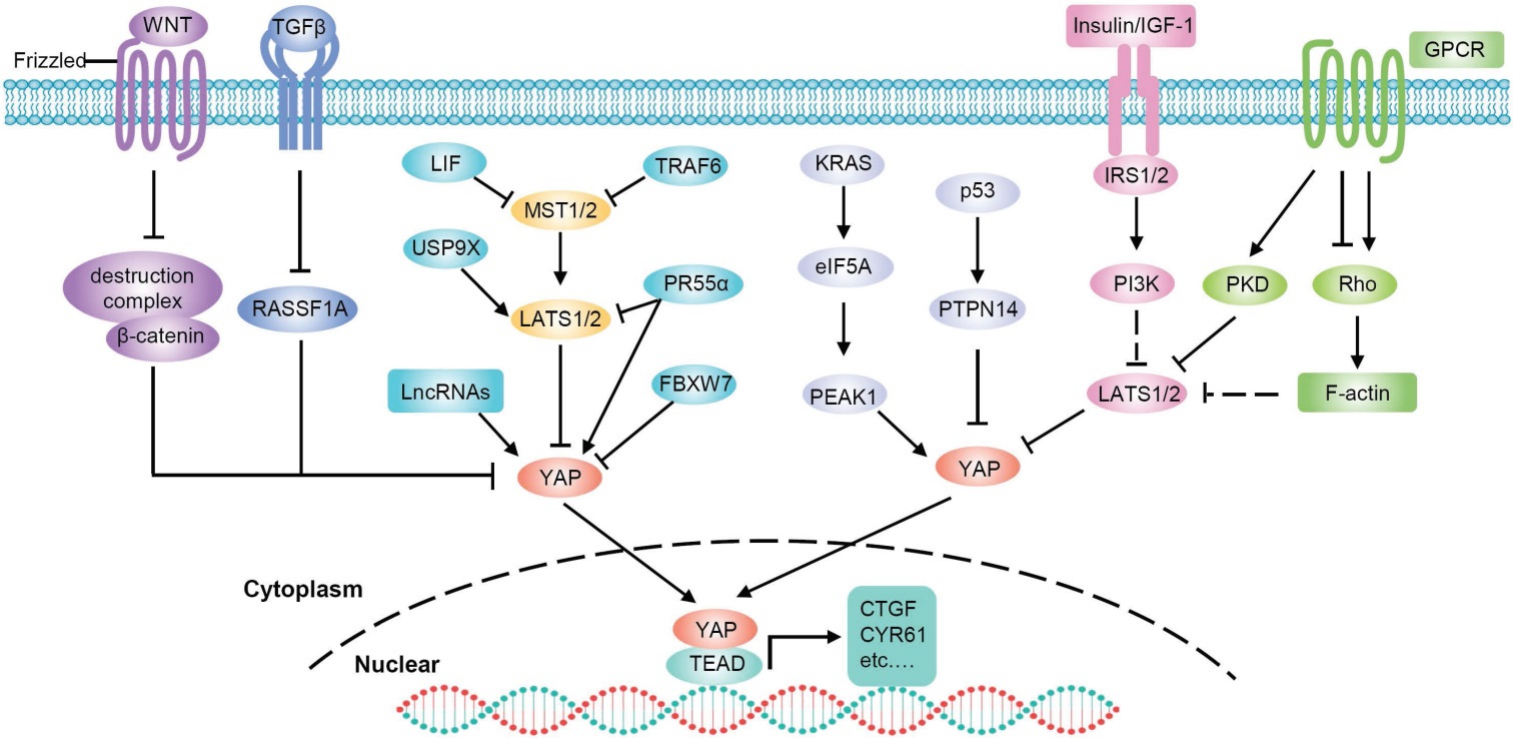
** Regulation of YAP in PDAC.** Multiple signals are integrated to regulate YAP activity. Soluble factors binding to GPCRs regulate LATS kinase through Rho. The insulin/IGF-1 receptor contributes to YAP activation through PI3K. WNT stimulation diverts YAP away from the β‑catenin destruction complex. TGF-β facilitates YAP nuclear translocation through RASSF1A. Other factors also affect YAP activity, in Hippo dependent or independent manners.

A complex network of upstream inputs, including cell polarity, mechanical stress, cell junctions and G-protein-coupled receptor (GPCR) signaling, modulates Hippo pathway activity 75. Hippo signaling can also crosstalk with other signaling pathways, such as transforming growth factor-β (TGFβ) 76, 77 and WNT/β-catenin pathways to modulate YAP activity 78-80. GPCRs function as key transducers of extracellular signals into the cell 81. Depending on the nature of downstream G proteins, GPCRs can either enhance or suppress YAP activity through LATS kinases 75. TGF-β facilitates YAP/SMAD2 nuclear translocation by targeting RASSF1A, which is the scaffold of Hippo pathway 77. WNT stimulation diverts YAP away from the β‑catenin destruction complex, causing YAP nuclear accumulation and β‑catenin stabilization 78. In addition, the Hippo pathway also crosstalks with other pathways, such as PI3K/AKT, Hedgehog, Notch, and mTOR 82-87, to modulate YAP activity. In this section, we will focus on the regulation of YAP in PDAC (**Figure 4**).

In human PDAC, the crosstalk between insulin/IGF-1 receptor and GPCR systems contributes to YAP activation through PI3K and PKD 88. In addition, YAP is a major mediator of the pro-oncogenic mutant p53 89 and p53 negatively regulates YAP through PTPN14 activation 90. Therefore, the tumor suppressor activity of p53 in pancreatic cancer is at least partially mediated via the inactivation of YAP 90. Moreover, in PDAC, mutant KRAS upregulates eukaryotic translation initiation factor 5A (eIF5A) and PEAK1, and eIF5A-PEAK1 signaling promotes YAP expression 91. PR55α, a PP2A regulatory subunit, has been reported to be essential for the tumor-promoting functions of YAP in pancreatic cancer. There are two pathways by which PR55α regulates YAP: activating YAP by inhibiting the MOB1-triggered autoactivation of LATS1/2 or directly interacting with YAP 92. Chen et al. identified an MST4-MOB4 complex, whose overall structure is similar to that of the MST1-MOB1 complex that can antagonize the MST1-MOB1 complex to promote YAP activity and play a pro-oncogenic role in pancreatic cancer 93. Glucose-regulated protein 78kDa on the surface of cancer cells (CS-GRP78) activates YAP in a Rho-dependent manner, promoting motility and radiation-resistance of PDAC cells 94. In addition, leukemia inhibitory factor (LIF) inhibits the activity of the Hippo-signaling pathway and subsequently increases YAP transcriptional activity in KRAS-driven pancreatic cancer. Therefore, blockade of LIF provides an attractive approach to improving therapeutic outcomes of pancreatic cancer 95.

Ubiquitylation is an important mechanism that regulates YAP activity through the proteasome degradation pathway 96-99. It was reported that the E3 ubiquitin ligase SIAH2 activates YAP by destabilizing LATS2 in response to hypoxia 96. Since hypoxia is a critical feature in PDAC, SIAH2 may also play an important role in pancreatic cancer through YAP. Moreover, deubiquitylase USP9X can suppress pancreatic cancer progression by inhibiting YAP through the deubiquitination and stabilization of LATS2 100, 101. FBXW7, which is a component of the Skp1-Cullin1-Fbox E3 ligase complex, has been identified as a potent suppressor of *Kras*^G12D^-induced pancreatic tumorigenesis. The anticancer effect of FBXW7 is, at least in part, due to its proteolytic regulation of YAP 102. TRAF6 activates YAP by promoting the degradation of MST1 12. Recently, studies have found that TAK1 prevents YAP degradation through a complex with TRAF6 and inhibition of TAK1 can significantly impair the oncogenic functions of YAP in pancreatic cancer 103.

LncRNAs are also involved in the regulation of YAP in pancreatic cancer. LncRNA UCA1 increases YAP nuclear localization and stabilization by forming shielding composites, thus promoting the migration and invasion of pancreatic cancer cells 104. LINC01559 accelerates pancreatic cancer cell proliferation and migration through a YAP-mediated pathway. On the one hand, LINC01559 serves as a ceRNA of YAP by sponging miR‐607; on the other hand, LINC01559 may inhibit YAP phosphorylation and enhance its activity by directly interacting with YAP 105. LncRNA THAP9-AS1 promotes PDAC growth and leads to a poor clinical outcome via regulating YAP 61. Mechanistically, THAP9-AS1 spongs miR-484, leading to YAP upregulation. In addition, THAP9-AS1 binds to YAP and inhibits the phosphorylation-mediated inactivation of YAP by LATS1. Reciprocally, YAP promotes THAP9-AS1 transcription to form a feed-forward circuit. Moreover, lncRNA MALAT1 and Linc-RoR have also been found to activate the Hippo/YAP pathway in PDAC cells 106, 107. Taken together, YAP is modulated by a complex signaling network and is commonly overactivated in pancreatic cancer cells.

## Therapeutic strategies targeting YAP

**Figure 5:**
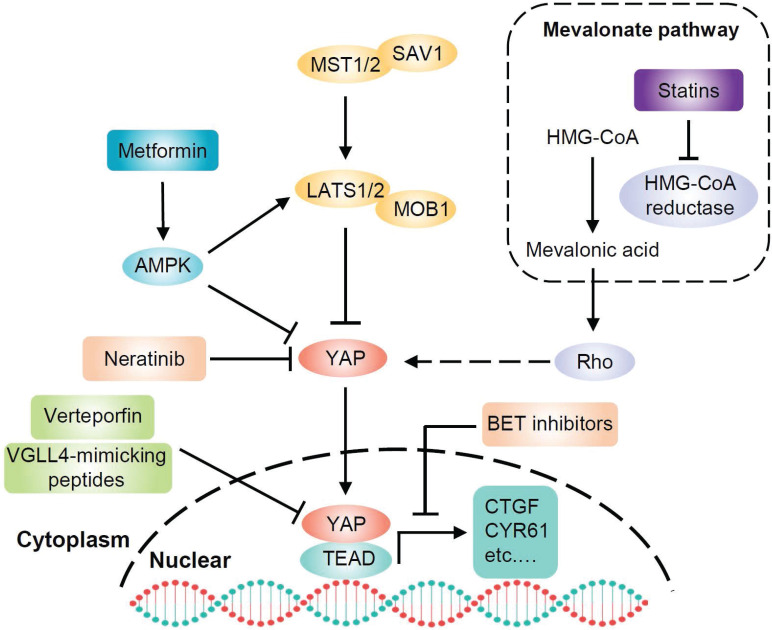
** Targeting YAP for PDAC therapy.** There are several strategies for targeting YAP in PDAC. Statin-mediated inhibition of HMG-CoA reductase in the mevalonate pathway reduces the geranylgeranylation and membrane localization of Rho GTPases, restricting YAP nuclear accumulation and thus its activity. Verteporfin and VGLL4-mimicking peptides disrupt the interaction between YAP and TEAD, inhibiting YAP-induced transcription. Metformin activates AMPK, which inhibits YAP both directly and by activating LATS kinases. BET inhibitors oppose the transcription of YAP-regulated genes. Neratinib increases the phosphorylation of LATS1 and YAP, causing YAP cytosolic accumulation and degradation.

*Kras* mutation is the most prominent driver of pancreatic cancer, and YAP is a critical KRAS effector. To date, KRAS is not a viable therapeutic target for pancreatic cancer. Even if KRAS could be effectively inhibited by new therapies, YAP amplification could provide a potential pathway for cancer recurrence. Therapeutically targeting YAP is clearly a very challenging yet exciting goal for PDAC. Studies have found novel approaches to inhibit YAP activity, including drugs such as verteporfin, statins and metformin (**Figure 5**).

### Verteporfin

Since YAP binds to TEAD transcription factors to induce target gene expression, designing compounds that directly dissociate YAP and TEAD interaction will be the most direct and effective approach to suppress YAP-induced oncogenic events 108. Verteporfin, a photosensitizer clinically used in photodynamic therapy, abrogates the interaction between YAP and TEAD, thereby inhibiting YAP transcriptional activity 109. Additionally, verteporfin has been clinically used for neovascular macular degeneration and exhibits limited toxicity, which provides an opportunity for its clinical application in cancer. Studies have demonstrated that verteporfin suppresses cell survival, angiogenesis and vasculogenic mimicry of PDAC. The mechanism accounting for these activities of verteporfin is to suppress the expression of targeted genes, such as cyclinD1, cyclinE1, Ang2, MMP2, VE-cadherin and a-SMA, by disrupting the YAP-TEAD complex 110. This study suggests that verteporfin may be a promising drug for pancreatic cancer by targeting YAP. Moreover, verteporfin can significantly enhance the antitumor efficacy of the pan-RAF inhibitor LY3009120 in pancreatic cancer by blocking the activation of alternative AKT signaling pathway after treatment with the RAF inhibitor 111. Therefore, the combination of verteporfin and pan-RAF inhibitors may be a potential approach for treating KRAS-mutant pancreatic cancer. In addition, another study has shown that verteporfin moderately enhances the antitumor activity of gemcitabine in a PDAC model by inhibiting autophagy, but its inhibitory effect on YAP was not evaluated in that study 112.

### Statins

Statins, inhibitors of the mevalonate pathway, are conventionally used to treat hypercholesterolemia and prevent cardiovascular disorders. Mechanistically, statins inhibit 3-hydroxy-3-methylglutaryl coenzyme A (HMG-CoA) reductase, which is the rate limiting enzyme in the generation of mevalonate 113. Mevalonate is a precursor of geranylgeranyl pyrophosphate, which is required for membrane localization and activation of Rho GTPases. Activated Rho plays a critical role in the nuclear accumulation of YAP through actin remodeling 34. Thus, statins have been identified as potential YAP inhibitors that oppose YAP nuclear localization and transcriptional responses by inhibiting the mevalonate pathway 114, 115. Preclinical studies have shown that statins delay the progression of pancreatic lesions to carcinoma or prevent the initial stages of PDAC in *Kras*^G12D^ mice 116, 117. It is also worth noting that substantial evidence indicates that statins have extensive tumor suppressor effects and accumulating clinical studies have shown a significant negative association between statins and cancer occurrence or survival 118.

### Metformin

Metformin, a widely administered drug for type 2 diabetes worldwide 119, activates AMPK, inhibits cell growth and reduces cancer occurrence 120. Moreover, the antidiabetic agent metformin has been found to inhibit YAP. Mechanistically, metformin activates AMPK, which in turn inhibits YAP via both LATS activation and direct YAP phosphorylation 41. Inhibition of YAP may contribute to the antitumor effect of metformin. Indeed, multiple epidemiological studies have shown that metformin is significantly associated with a reduced risk of cancer, especially pancreatic cancer, in diabetic patients 121-123. Another study found that oral administration of metformin strikingly decreases the incidence of PDAC in *Kras*^G12D^ mice with diet-induced obesity 124. This effect is associated with an increase in pancreatic AMPK activity and a decrease in pancreatic YAP expression. Furthermore, metformin can inhibit pancreatic cancer through other AMPK-mediated signaling pathways. For example, metformin inhibits pancreatic cancer growth by disrupting the crosstalk between GPCR and insulin receptor signaling systems 125. These results raise the possibility that metformin may be a potential therapeutic strategy for human pancreatic cancer.

### Other strategies

Bromodomain-containing protein 4 (BRD4) is a coactivator of the bromodomain and extraterminal domain (BET). A recent study has demonstrated that the transcriptional addiction in cancer cells is mediated by YAP through BRD4. Mechanistically, BRD4 interacts with YAP and is recruited to chromatin, enhancing the expression of a host of growth-regulating genes. The BET inhibitor JQ1 opposes the activity of BRD4 and downregulates the expression of YAP-regulated genes 126. These findings suggest the potential of BET inhibitors to target YAP, but the effect of these inhibitors in pancreatic cancer remains to be investigated. Neratinib is an irreversible ERBB1/2/4 inhibitor, which can downregulate the expression of other RTKs and mutant RAS proteins. Prior studies have demonstrated that neratinib causes mutant KRAS to localize in intracellular vesicles, concomitant with its degradation. Moreover, neratinib increases the phosphorylation of LATS1 and YAP, causing the majority of YAP to be translocated into the cytosol and degraded. Therefore, neratinib can coordinately suppress the functions of both mutant KRAS and YAP to kill pancreatic cancer cells 127. In addition, a peptide mimicking the function of VGLL4 acts as a YAP antagonist and potently suppresses tumor growth 128, suggesting that the VGLL4-mimicking peptide may be a promising therapeutic strategy against YAP-driven human cancers, however, its effect in pancreatic cancer has not been revealed.

## Conclusions and further perspectives

Despite the major advances in revealing the molecular mechanism driving PDAC, this disease remains the most lethal human cancer. Almost all PDACs harbor *Kras* mutation, which is necessary but not completely sufficient for the development of invasive PDAC. YAP has been identified as a critical effector downstream of KRAS, but its activation enables bypass of KRAS addiction in pancreatic cancer. There is no doubt that YAP plays a critical role in cancer development and is emerging as an attractive therapeutic target for PDAC. However, some challenges remain unresolved. First, it is not fully elucidated what triggers the activation of YAP, since the core components of the Hippo pathway are typically not mutated in PDAC. Second, the downstream effectors and mechanisms that mediate the function of YAP remain incompletely elucidated. Importantly, more efforts are needed to develop effective inhibitors that directly target YAP or dissociate the YAP-TEAD interaction. These drugs can be used alone or in combination with other therapies, such as chemotherapy, radiotherapy and immunotherapy, for more effective treatment of advanced PDAC. Finally, it is also essential to develop biomarkers, especially noninvasive biomarkers, to accurately select patients for YAP-targeted therapy, and to facilitate the real-time evaluation of therapeutic effects.

## Abbreviations

ADM: acinar to ductal metaplasia
BET: bromodomain and extraterminal domain
BRD4: Bromodomain-containing protein 4
CSC: cancer stem cell
DGUOK: deoxyguanosine kinase
eIF5A: eukaryotic translation initiation factor 5A
EMT: epithelial-mesenchymal transition
GLUT3: glucose-transporter 3
GPCR: G-protein-coupled receptor
LATS1/2: large tumor suppressor 1/2
LIF: leukemia inhibitory factor
MDSC: myeloid-derived suppressor cells
MST1/2: mammalian Ste20-like kinases 1/2
OGT: O-GlcNAc transferase
PDAC: pancreatic ductal adenocarcinoma
PFK1: phosphorfructokinase
SCD1: stearoyl-CoA-desaturase 1
TAZ: transcriptional co‑activator with PDZ-binding motif
TEAD: TEA domain
TGFβ: transforming growth factor-β
VGLL4: vestigial-like protein 4
YAP: Yes-associated protein
